# Supplementary material for: Reversible Modulation of Motile Cilia by a Benzo[e][1,2,4]triazinone: A Potential Non-Hormonal Approach to Male Contraception
Source: Cells. 2025 May 9;14(10):688. doi: 10.3390/cells14100688 (PMC12110593; doi:10.3390/cells14100688)
Supplement: Supplementary file 1 [file cells-14-00688-s001.zip › cells-3582546-supplementary.pdf]

A.

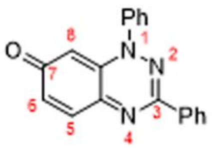


1

B.

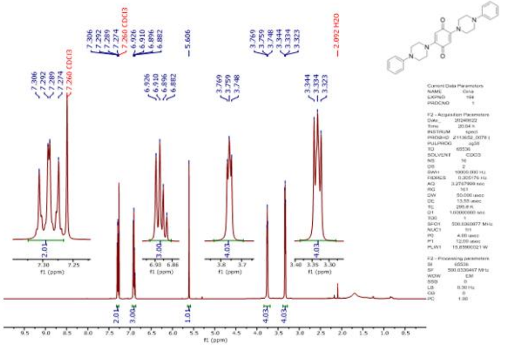

C.

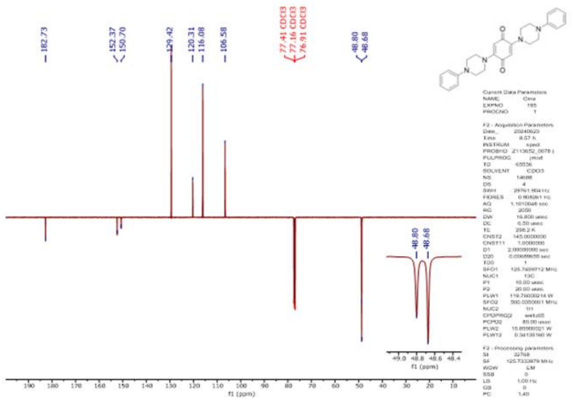


**Figure S1:**

A. Chemical structure of 1,3-diphenylbenzo[e][1,2,4]triazin-7-one (**1**) showing IUPAC numbering of the core ring system.

B. $^{1}$H NMR of 2,5-bis(4-phenylpiperazin-1-yl)cyclohexa-2,5-diene-1,4-dione (**26**) in $CDCl_3$.

C. $^{13}$C NMR of 2,5-bis(4-phenylpiperazin-1-yl)cyclohexa-2,5-diene-1,4-dione (**26**) in $CDCl_3$.

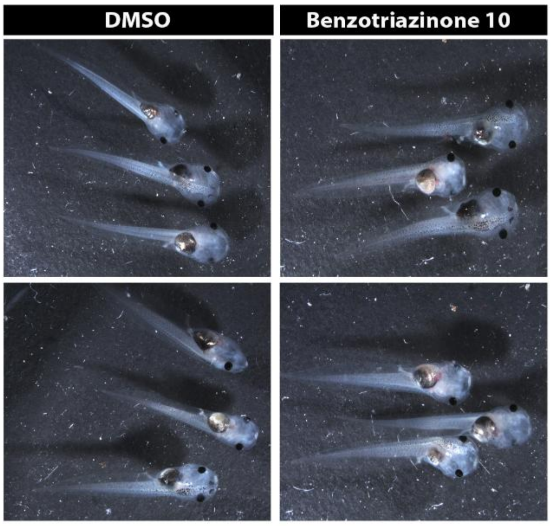


**Figure S2: DFF10 does not affect development of *Xenopus* embryos**

Brightfield images of *Xenopus* embryos treated with DMSO or benzotriazinone **10**, showing no apparent morphological differences between groups, indicating that benzotriazinone **10** does not cause gross developmental defects

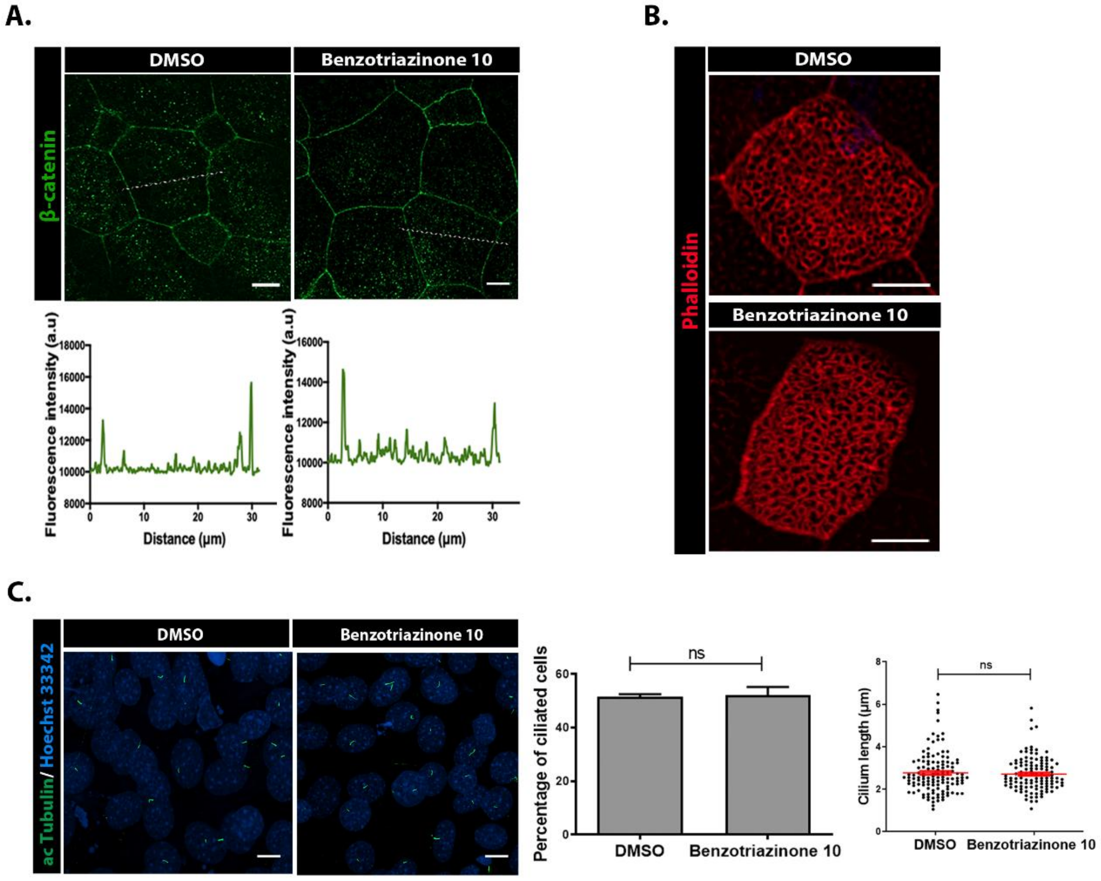


**Figure S3: Benzotriazinone 10 does not affect apical actin network**

A. Immunofluorescence images of β-catenin in *Xenopus* epithelial tissue treated with DMSO or benzotriazinone **10**, demonstrating that benzotriazinone **10** does not disrupt tissue morphology or apical cell-cell junctions. Fluorescence intensity plots (bottom) along the dashed white lines show comparable β-catenin distribution between treatments. Scale bars: 10 µm.
B. Immunofluorescence images of Phalloidin in DMSO and benzotriazinone **10**-treated MCCs. Scale bars, 10 µm.
C. Immunofluorescence analysis of primary cilia from DMSO- and benzotriazinone **10**-treated NIH3T3 cells. Acetylated tubulin marks cilia, and nuclei are counterstained with Hoechst 33342 (blue). Quantification of the percentage of ciliated cells and cilium length demonstrates no differences between DMSO- (n=125) and benzotriazinone **10**-treated (n=135) groups. Scale bars = 5 µm.

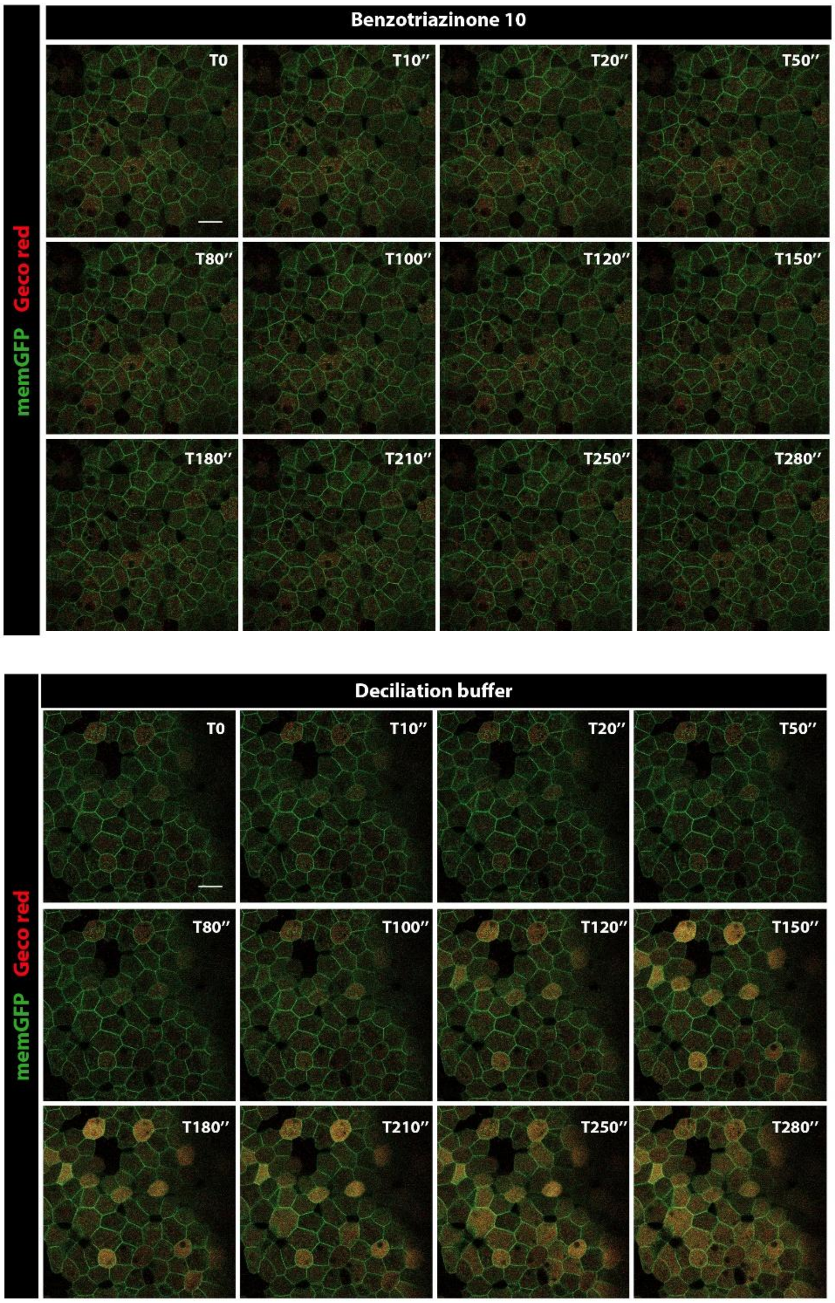


**Figure S4: Intracellular Ca2+ levels remain unaffected upon benzotriazinone 10 treatment**

Stills from time-lapse movies showing MCCs expressing the calcium ion indicator GECO-RED and memGFP to visualize ciliary axonemes. At T80'' benzotriazinone **10** or deciliation buffer was added into the media of embryos.

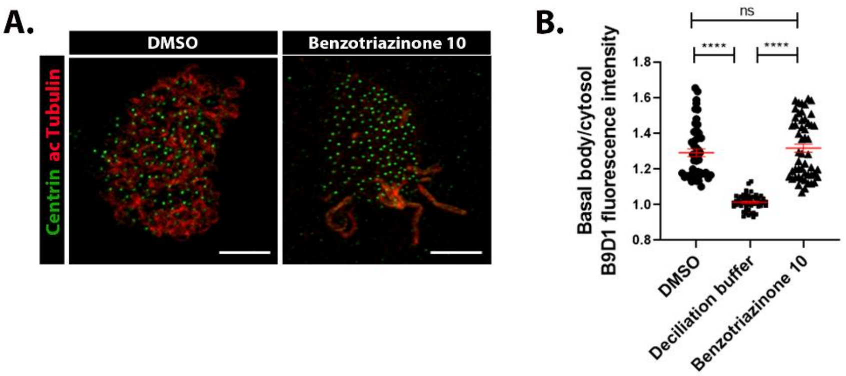


**Figure S5**:

A. Immunofluorescence images of Centrin, a marker of basal bodies in DMSO- and benzotriazinone **10**-treated MCCs. Scale bars, 10 µm.
B. Quantification of fluorescence intensity of mEmerald B9D1 at the basal bodies normalized to the cytosol of MCCs (n=49 basal bodies of DMSO-treated MCCs, n=51 basal bodies of deciliation buffer-treated MCCs, n=52 basal bodies of benzotriazinone **10**-treated MCCs, ****p<0.0001.
